# Supplementary material for: Comparative Interactome Analysis of Emerin, MAN1 and LEM2 Reveals a Unique Role for LEM2 in Nucleotide Excision Repair
Source: Cells. 2020 Feb 18;9(2):463. doi: 10.3390/cells9020463 (PMC7072835; doi:10.3390/cells9020463)
Supplement: Supplementary file 1 [file cells-09-00463-s001.zip › supplementary material/Supplementary Material.pdf]

Supplementary Material:

Supplementary Table S1: Mass spectrometry data. Interactors detected in LEM2 (LEM2), MAN1 (LEMD3) emerin (EMD) and GFP (GFP) BioID. A) Raw spectral count data MS analyses. B-H): High confidence (hc) (average probability (AvgP) of ≥ 0.45) interactors. Bait: BirA*-Emerin-V5 (EMD), BirA*-LEM2-V5 (LEM2) and BirA*-MAN1-V5 (LEMD3), Protein GI (NCBI): Protein GenInfo Identifier from (National Center for Biotechnology), PreyGene: Gene name, Spec: Spectral counts of each BioID experiment, SpecSum: sum of spectral counts, AvgSpec: average spectral counts per BioID experiment, NumRep: Number of BioID experiments, iProp: individual SAINT probabilities, ctrlCounts: spectral counts of all BioID controls, AvgP: average SAINT score (probability score which shows the average of all individual SAINT probabilities), MaxP: largest probability (iProb) (reports the largest probability of a bait-prey pair across all replicates), FDR: false discovery rate (post-hoc calculation of the false discovery rates of AvgP).

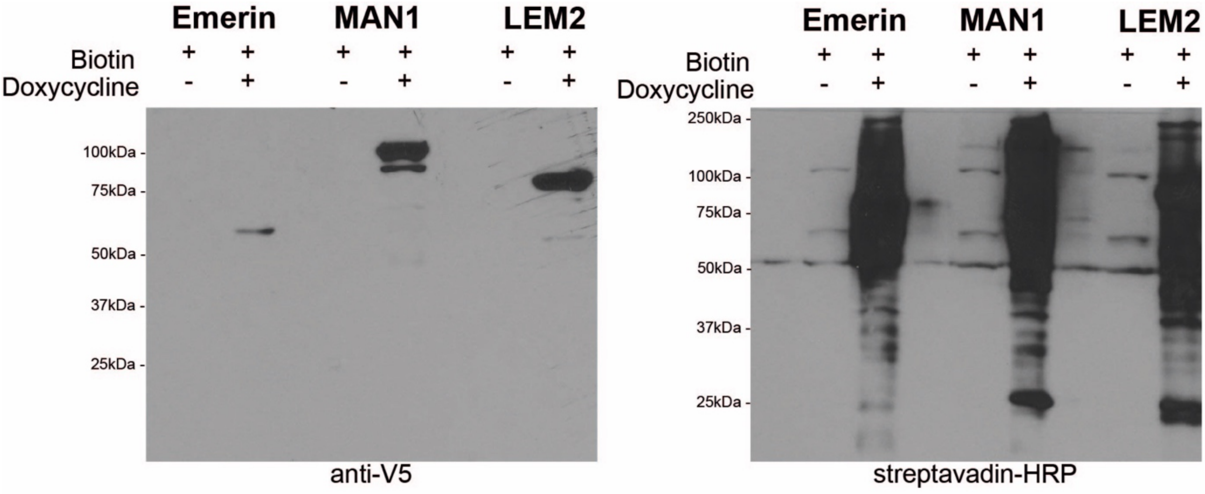

Supplementary Figure 1: Full unedited immunoblot for Figure 1D.
